# High Temperature Deformation Mechanism in Hierarchical and Single Precipitate Strengthened Ferritic Alloys by In Situ Neutron Diffraction Studies

**DOI:** 10.1038/srep45965

**Published:** 2017-04-07

**Authors:** Gian Song, Zhiqian Sun, Lin Li, Bjørn Clausen, Shu Yan Zhang, Yanfei Gao, Peter K. Liaw

**Affiliations:** 1 Department of Materials Science and Engineering, The University of Tennessee, Knoxville, TN, 37996-2200, USA; 2 Lujan Center, Los Alamos National Laboratory, Los Alamos, NM, 87545, USA; 3 ISIS, Science and Technology Facilities Council, Rutherford Appleton Laboratory, Chilton, Didcot, Oxfordshire OX11 0QX, UK; 4 Materials Science and Technology Division, Oak Ridge National Laboratory, Oak Ridge, TN, 37831, USA

## Abstract

The ferritic Fe-Cr-Ni-Al-Ti alloys strengthened by hierarchical-Ni_2_TiAl/NiAl or single-Ni_2_TiAl precipitates have been developed and received great attentions due to their superior creep resistance, as compared to conventional ferritic steels. Although the significant improvement of the creep resistance is achieved in the hierarchical-precipitate-strengthened ferritic alloy, the in-depth understanding of its high-temperature deformation mechanisms is essential to further optimize the microstructure and mechanical properties, and advance the development of the creep resistant materials. In the present study, *in*-*situ* neutron diffraction has been used to investigate the evolution of elastic strain of constitutive phases and their interactions, such as load-transfer/load-relaxation behavior between the precipitate and matrix, during tensile deformation and stress relaxation at 973 K, which provide the key features in understanding the governing deformation mechanisms. Crystal-plasticity finite-element simulations were employed to qualitatively compare the experimental evolution of the elastic strain during tensile deformation at 973 K. It was found that the coherent elastic strain field in the matrix, created by the lattice misfit between the matrix and precipitate phases for the hierarchical-precipitate-strengthened ferritic alloy, is effective in reducing the diffusional relaxation along the interface between the precipitate and matrix phases, which leads to the strong load-transfer capability from the matrix to precipitate.

Coherent precipitate-strengthened alloys in Fe-Cr-Ni-Al system have received attentions as a candidate for high-temperatures applications, such as fossil-power plants, due to their promising mechanical properties, corrosion/oxidation resistance, and cost efficiency^1^,^2^,^3^,^4^,^5^,^6^. These materials consist of the primary B2-NiAl precipitate homogeneously distributed in the body-centered cubic (bcc) Fe matrix. The small difference in the lattice parameters between the B2-NiAl (ordered bcc) and bcc-Fe (disordered bcc) gives rise to a small lattice mismatch and coherent interface with a small elastic strain^7^,^8^, which is known to be beneficial to the resistance to the coarsening behavior of the precipitate during the long-term exposure to high-temperatures, as in the classic γ/γ′ superalloys^9^,^10^,^11^,^12^,^13^,^14^.

Recently, two-phase NiAl/Ni_2_TiAl-precipitate and single-phase Ni_2_TiAl-precipitate-strengthened ferritic alloys have been developed via the addition of Ti to the NiAl-precipitate strengthened ferritic alloys^15^,^16^,^17^,^18^. The microstructures of the alloys, such as chemistry and morphology of the constitutive phases within the precipitates, have been systematically characterized using transmission-electron microscopy (TEM) and atom-probe tomography (APT)^18^,^19^,^20^. The microstructural features of the 4-wt.% and 2-wt.%-Ti alloys aged at 973 K for 100 hours are displayed in Fig. 1. Based on the APT and TEM results, the single-phase Ni_2_TiAl-precipitate-strengthened ferritic alloy (SPSFA) is hardened by single L2_1_-Ni_2_TiAl precipitates in the bcc-Fe matrix^15^,^18^. The two-phase NiAl/Ni_2_TiAl-precipitate-strengthened ferritic alloy is reinforced by parent L2_1_-Ni_2_TiAl precipitates, which is further divided by sub-structures of the B2-NiAl phase^18^. Such a two-phase precipitate-strengthened ferritic alloy has been described as hierarchical-precipitate-strengthened ferritic alloy (HPSFA)^18^, characterized by the relative chemical ordering, spatial dimensions of the constitutive phases, and their spatial distribution^17^. Both alloys often contain a small Fe inclusion within the precipitates, which was previously determined by energy-dispersive X-ray spectroscopy^18^,^19^. It was suggested that the hierarchical precipitate structure is effective to retain the coherent interface, whereas the single Ni_2_TiAl precipitate tends to form misfit dislocation at the interface in order to accommodate the large lattice mismatch^18^,^20^.

In the previous paper, it was shown that the creep resistance of HPSFA and SPSFA is significantly improved, as compared to the NiAl-strengthened ferritic alloys^18^. For instance, the secondary creep rates of these alloys at 973 K are significantly reduced by more than four orders of magnitude, as compared to a NiAl-strengthened ferritic alloy^4^ and conventional ferritic steels^21^,^22^,^23^,^24^. Moreover, HPSFA shows much better creep resistance than SPSFA with a higher volume fraction of the precipitates^18^. It was suggested that the elastic strain field in the matrix created by misfitting precipitates of HPSFA plays a critical role in enhancing the elastic interaction between the precipitate and mobile dislocations, and, hence, the creep resistance.

In the present study, we have characterized *in*-*situ* dynamic evolution of elastic/plastic response of constitutive phases in hierarchical- and single-precipitate-strengthened ferritic alloys during tensile/relaxation deformation at 973 K, by state-of-the-art neutron diffraction at the Spallation Neutron Source, Los Alamos National Laboratory and Rutherford Appleton Laboratory, and crystal-plasticity finite-element model. The *in*-*situ* neutron diffraction technique has been extensively utilized to understand the governing deformation mechanisms of engineering materials, such as evolution of elastic strains and interactions (load-sharing) between constitutive phases/intergranular grains^25^,^26^,^27^,^28^,^29^. The experimental and simulated elastic/plastic response of the matrix/precipitate phases during tensile deformation demonstrates that the coherent elastic-strain field in the matrix, reinforced by the hierarchical-precipitate structure, improves the load-transfer capability from the matrix to precipitate, and, thus, the high-temperature mechanical properties. The response to stress relaxation reveals that the hierarchical-structure-enhanced elastic field is effective in reducing the diffusional relaxation along the interface between the precipitate and matrix phases.

## Results

**Figure 1:**
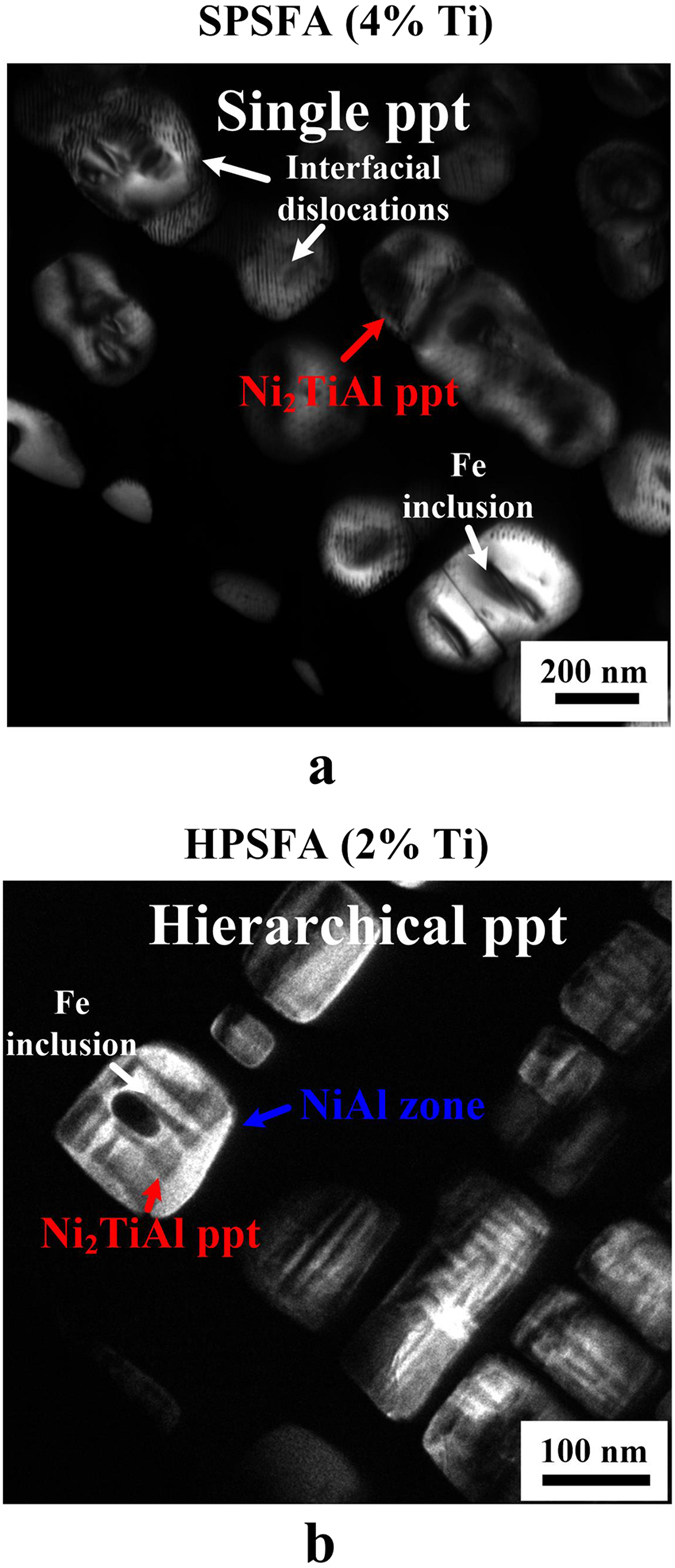
DF-TEM images of the precipitates-strengthened ferritic alloys. Dark-field (DF) transmission-electron-microscopy (TEM) images showing the microstructures of (**a**) SPSFA, and (**b**) HPSFA (ppt stands for precipitate).

The precipitates in HPSFA and SPSFA show apparent microstructural differences in terms of the size, morphology, and interface structure of the precipitates in Fig. 1. The single L2_1_-type precipitate in SPSFA with an average size of 220 ± 46 nm^18^ is decorated with a high density of interfacial (misfit) dislocations, which indicates the high level of the lattice mismatch between the Fe and L2_1_ phases. The misfit dislocations are formed to release the elastic strain of the precipitate, which result in a change from cuboidal to spherical shapes^18^. That is, with the elastic strain energy being dominant, the precipitate shape tends to take a form that resembles the elastic stiffness anisotropy – being cuboidal in cubic crystals. In contrast, the hierarchical precipitate with an average width of 111 ± 27 nm^18^ is free of the interfacial dislocation, which reflects the coherent interface, resulting from the relatively-small lattice mismatch between the matrix and precipitate, as compared to that of SPSFA. Moreover, the cuboidal morphology of the hierarchical precipitate indicates the high level of the elastic strain of the precipitate. Based on the precipitate features, such as the morphology of the precipitates and coherent/semi-coherent interfaces in HPSFA and SPSFA, it is believed that the magnitude of the elastic strain field in the matrix of HPSFA is greater than that of SPSFA^18^.

**Figure 2:**
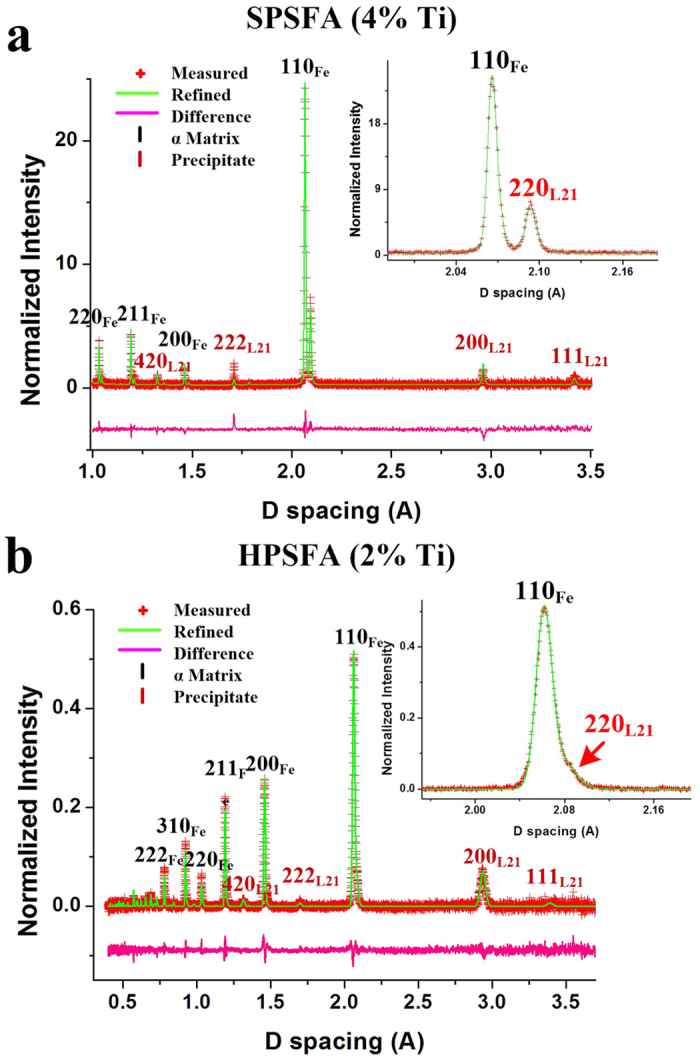
Comparison of the ND results between SPSFA and HPSFA. Representative neutron-diffraction patterns of (**a**) SPSFA and (**b**) HPSFA measured at room temperature without loading. Enlarged patterns in (**a**) and (**b**) clearly exhibit well-separated and overlapped fundamental (110)_Fe_ and (220)_L21_ peaks for SPSFA and HPSFA, respectively. The red cross represents the measured data. The green curve is the fitted profile using the GSAS Rietveld analysis^30^. The pink curve presents the difference between the fitted profile and measured data.

**Figure 3:**
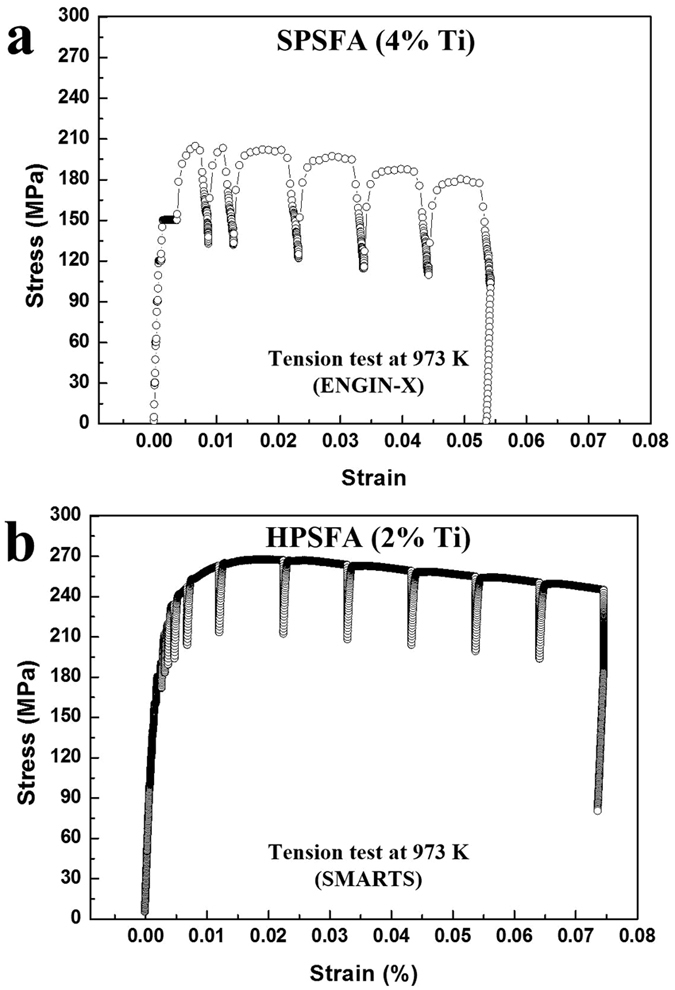
Macroscopic stress-strain curves. Stress-strain curves recorded during *in*-*situ* tensile experiments at 973 K for (**a**) SPSFA and (**b**) HPSFA.

Representative neutron-diffraction (ND) patterns measured at room temperature without loading (a reference state, 5 MPa) for SPSFA and HPSFA are presented in Fig. 2, with the refined profile by the General Structural Analysis System (GSAS) Rietveld analysis^30^. Both alloys show the fundamental reflections (110, 200, 211, and 220), and super-lattice reflections (111, 200, 222, and 420). The superlattice peaks of (111) and (222) are unique to the L2_1_ structure, as compared to the B2 structure, which supports the formation of the L2_1_ structure in both alloys. The enlarged patterns in the insets of Figs 2a and 3b clearly show the well-separated fundamental 110_Fe_/220_L21_ peaks for SPSFA and overlapped peaks for HPSFA, respectively. This trend supports the TEM observation indicating that the lattice misfit between the α-Fe and L2_1_ phases of SPSFA is greater than that of HPSFA. The averaged lattice parameters of the α-Fe and L2_1_ phases, determined by GSAS Rietveld analysis^30^, and corresponding lattice misfit, defined as Eq. [8] (See Methods Section: Neutron Data Analysis), for SPSFA and HPSFA at room temperature and 973 K are summarized in Table 1. The lattice misfit of SPSFA (1.32%) is greater than that of HPSFA (0.77%) at 973 K, and the larger lattice misfit causes the formation of the misfit dislocations at the interface between the Fe matrix and L2_1_ precipitate for SPSFA (Fig. 1a). In contrast, the lower lattice misfit of HPSFA leads to the retention of the coherent interface, as reflected by the TEM observations in Fig. 1b.

Figure 3a and b show the macroscopic stress versus strain curves during *in*-*situ* tension loading experiments on SPSFA and HPSFA at 973 K, respectively. The creep strain during the load-holding period is not significant when the stress is less than the macroscopic yield strength. Since extensive creep strain occurs at stresses close to or higher than the yield strength^31^, the displacement or strain gauge holding mode were used to measure the ND data at the higher stresses. During yielding, both alloys exhibit a short hardening stage, and the hardening behavior of HPSFA seems to be more significant than that of SPSFA. The 0.2% macroscopic yield strengths for SPSFA and HPSFA were estimated to be about 200 MPa and 230 MPa, respectively. Once the applied stress reaches the highest level (ultimate tensile stress; UTS), the stress softening, where the stress gradually decreases with increasing the plastic strain, occurs for both alloys. During the ND measurements in the plastic regimes (displacement or strain gauge holding), a decrease in the applied stress is observed (stress relaxation), and the amount of the stress decay seems to depend on the tested alloys.

**Figure 4:**
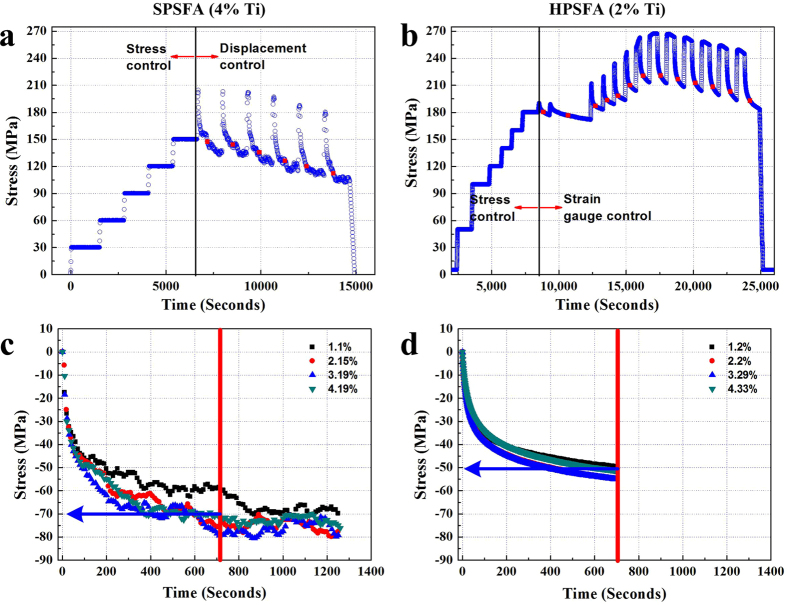
Temporal evolution of macroscopic stress. Temporal evolution of macroscopic stress for (**a**) SPSFA and (**b**) HPSFA during the whole *in*-*situ* tension experiments at 973 K. Temporal evolution of relative stress relaxation for (**a**) SPSFA and (**b**) HPSFA during ND measurements with respect to plastic strain.

Figure 4 shows the temporal evolution of the applied stress in the plastic deformation region (Fig. 3) where the ND measurements were carried out with the displacement or strain gauge holding mode. The averaged stress was estimated during each holding step, and marked as red squares in Fig. 4a and b. Note that the holding time (about 20 mins) of SPSFA for the ND measurements is longer than that of HPSFA (about 12 mins). This is because the holding time was required to obtain the statistically-reliable ND data in each ND instrument. Figure 4c and d show the temporal evolution of the relative stress decay with different plastic strains. It can be seen that the total amount of the stress decay in each alloy is independent upon the plastic strains considered. The stress relaxation curve can be divided into three regimes, initial rapid decline, transition, and asymptotic regions, as observed in stress relaxation experiments at high temperatures^32^,^33^. Figure 4c and d provide the insight into the efficiency of the stress relaxation varying with the tested alloys. For example, the total stress decay of SPSFA is greater than that of HPSFA. The magnitude of the stress decay of SPSFA for 700 seconds is about 70 MPa, which is about 20 MPa higher than that of HPSFA, as indicated by blue arrows in Fig. 4c and d. This suggests that the stress relaxation occurs more easily for SPSFA at 973 K, as compared to HPSFA.

**Figure 5:**
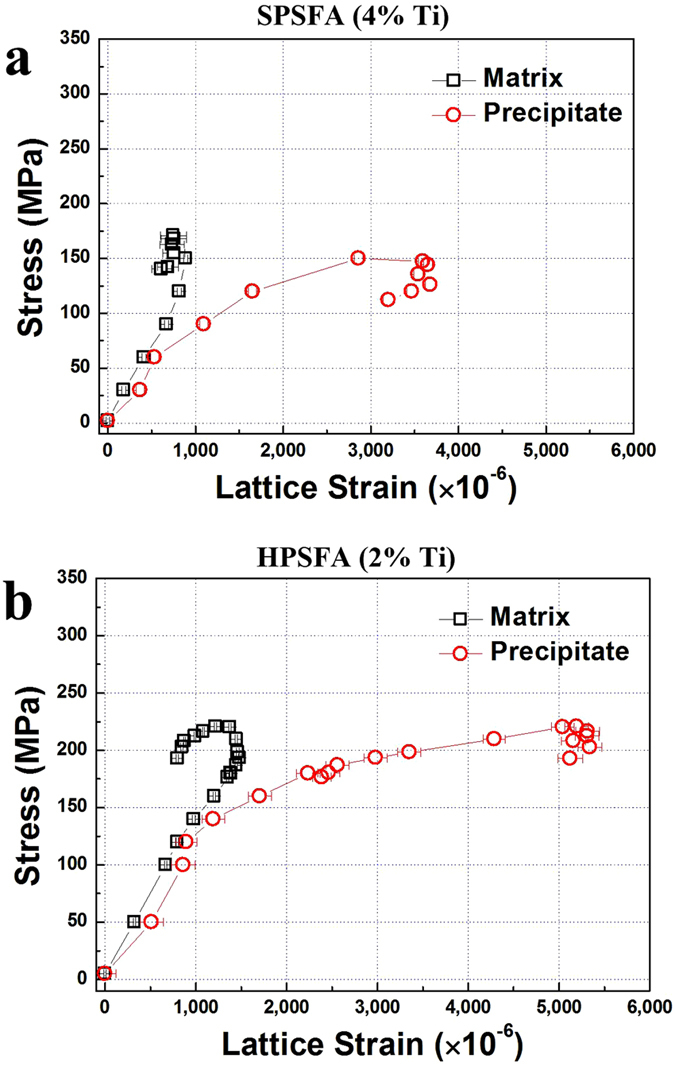
Lattice-strain evolution from the *in*-*situ* tension ND experiments. Average phase strains along the axial direction at 973 K as a function of average stress during the *in*-*situ* tension experiments on (**a**) SPSFA and (**b**) HPSFA.

The evolution of the average-phase elastic-strains of the matrix and precipitate as a function of average stress at 973 K are displayed in Fig. 5. Note that the average stress estimated during each holding step in Fig. 4a and b is employed in the curves. Similar to the macroscopic stress-strain curves (Fig. 3), the elastic strain evolution for both alloys exhibits the two regimes; elastic and plastic regimes. Specifically, the elastic region is characterized by the linear slope of the lattice strain with respective to the average stress. The onset of the plastic deformation is marked by the deviation from the linear response on the elastic strain-stress curves. For example, the slope of the elastic strain of the matrix is increased relative to the linear elastic-response, which indicates that the matrix cannot assume the further elastic strain and begins to plastically deform^34^. Therefore, the yielding of the matrix leads to the plastic strain besides the elastic strain. In contrast, the reduced slope of the elastic strain of the precipitate reflects that the precipitate is still deforming elastically, and the additional plastic strain from the matrix is transferred to the precipitate^29^, which induces the decrease in the slope of the elastic strain of the precipitate. The microscopic yielding occurs around the average stress of 150 MPa for SPSFA and 180 MPa for HPSFA, respectively. After the matrix is yielded, the elastic strain of the precipitate increases with the average stress. The maximum elastic strain of the precipitate of HPSFA (about 5,000 μe) is greater than that of SPSFA (about 3,500 μe), which indicates the effectiveness of the load carrying capability of the precipitate in HPSFA than SPSFA. However, once the average stress reaches the maximum value, the elastic strain of the precipitate for both alloys remains fairly unchanged, while that of the matrix gradually decreases. This behavior corresponds to the softening behavior in macroscopic strain-stress curves (Fig. 3), where the applied stress slightly decreases with increasing the plastic strain.

**Figure 6:**
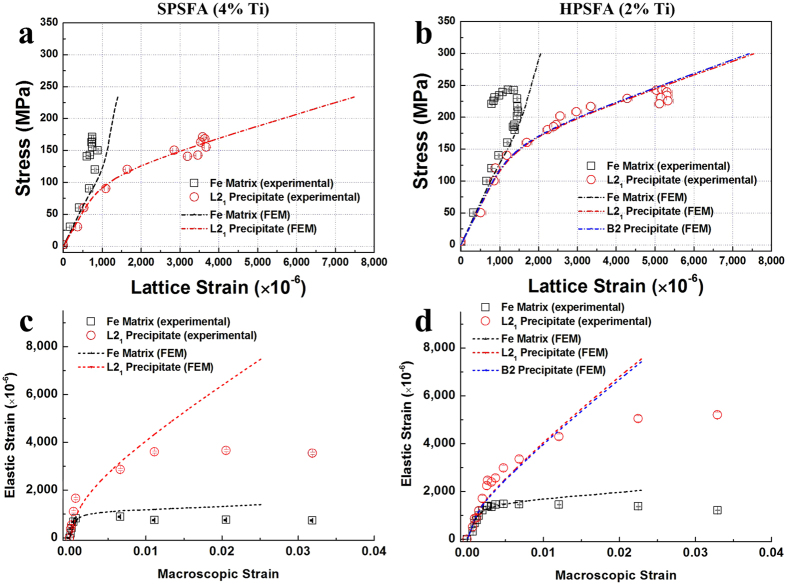
Comparison of lattice-strain evolution between the *in*-*situ* tension ND experiments and CPFEM. Average phase strains along the axial direction as a function of stress during tension deformation for (**a**) SPSFA and (**b**) HPSFA. The evolution of the average strain with respective to the macroscopic strain for (**c**) SPSFA and (**d**) HPSFA. Symbols are the experimental data, while lines are the simulated results.

The evolution of the averaged elastic phase strain as a function of stress was simulated using crystal-plasticity finite-element modelling (CP-FEM) for SPSFA and HPSFA. Figure 6 shows the CP-FEM results with the experimental measurements during the *in*-*situ* loading tests at 973 K. The dashed lines in Fig. 6 are the predictions, while the symbols are the experimental data. Note that both predicted and experimental data are the averaged phase strain determined from the Rietveld analysis. The CP-FEM results are qualitatively consistent with the experimental data in terms of the elastic-plastic transition and subsequent load transfer from the matrix to precipitate. Specifically, once the matrix is yielded, the elastic strain of the matrix is saturated, and the additional load from the matrix is transferred to the precipitate, leading to the observed splitting of the lattice strain curves. For HPSFA, the modeled evolution of the elastic strain for the B2 and L2_1_ phases within the precipitate is comparable to each other, and the B2 and L2_1_ phases assume the additional load from the plastically deforming matrix, which is similar to the evolution of the single L2_1_ precipitate in SPSFA. It seems that such a hierarchical structure within the precipitate has no considerable influence in terms of the microscopic load-transfer behavior. The strain hardening of HPSFA is observed to be more pronounced than that of SPSFA, as supported by the hardening parameters (h_0_; 500 for HPSFA, 100 for SPSFA) employed in the CP-FEM simulations (Table 2). The hardening parameter is related to the strain-hardening capability of the matrix according to Eq. [10] (See Methods Section: Crystal-Plasticity Finite Element Model), which indicates that the HPSFA has more strain-hardening capability of the matrix, as compared to SPSFA.

The simulated elastic strain of the matrix and precipitate continue to increase with the applied stress in Fig. 6a and b, which is not consistent with the experimental results. This is because the strain-softening occurs at 973 K after the applied stress reaches UTS (Fig. 3). Figure 6c and d show the simulated and experimental evolution of the elastic strain as a function of plastic strain. The simulated evolution tends to show the strain hardening behavior as the plastic strain increases, while the experimental evolution remains fairly constant after the plastic strain of about 1.0%. This discrepancy is believed to result from the diffusional process, such as dislocation and diffusional creep, which is not considered in the FEM simulations, and will be discussed in Discussion Section: Effect of Interface Structure on Mechanical Properties.

## Discussion

### Deformation Mechanism at 973 K

The 2-wt.%-Ti alloy is reinforced by the coherent precipitate with the hierarchical structure of the B2 and L2_1_ phases. The estimated macroscopic yield strength of HPSFA at 973 K is about 230 MPa, which is higher than that (200 MPa) of the 4-wt.%-Ti alloy strengthened by the semi-coherent single L2_1_ precipitate. It is well known that the yield strength for coherent precipitate-reinforced materials is controlled by shearing or Orowan dislocation bypass mechanisms^35^,^36^. It has been reported that the operational mechanisms are strongly dependent upon the size of the coherent/shearable precipitate^37^,^38^. In general, the shearing mechanism is dominant when the precipitate is smaller than a critical size of the precipitate, while the Orowan dislocation bypass mechanism is operational at the bigger size. The shearing mechanism involves three strengthening contributions, such as, ordering strengthening (Δ*σ*_1_), coherency strengthening (Δ*σ*_2_), and modulus mismatch strengthening (Δ*σ*_3_)^39^, and defined as,


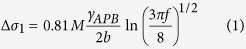



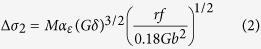



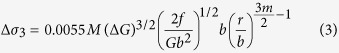


where *M* = 2.9 is the mean orientation factor for a bcc matrix^40^, b = 0.250 nm is the magnitude of the matrix Burgers vector, determined by the current ND results, f is the volume fraction of the precipitates, *G* = 57.0 GPa is the shear modulus of polycrystalline α-Fe at 973 K^41^, *α*_*ε*_ = 2.6 is a constant^39^, *δ* is the constrained lattice parameter mismatch, *r* is the mean precipitate radius, *m* is a constant taken to be 0.85^39^, and Δ*G* is the shear modulus mismatch between the matrix and the precipitates. The strength increase from the shear mechanism (Δ*σ*_1_, Δ*σ*_2_, and Δ*σ*_3_) is mainly influenced by the anti-phase boundary (APB) energy and size and volume fraction of the coherent precipitate.

**Figure 7:**
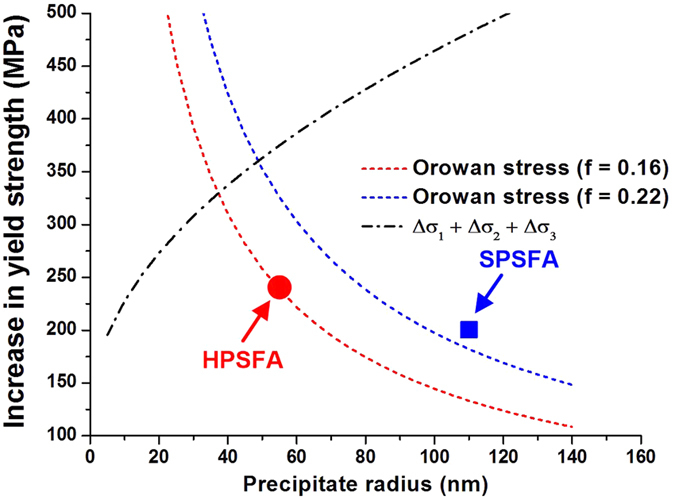
Strengthening contributions. Increase in yield stress as a function of precipitate radius at 973 K. Experimental points are obtained from the 0.2% yield stress measurements (Fig. 4), and the theoretical lines are calculated from Eqs [1, 2, 3, 4, 5] for the Orowan stress (*σ*_*OR*_) and shearing stress due to the ordering (Δ*σ*_1_), lattice mismatch (Δ*σ*_2_), and modulus mismatch (Δ*σ*_3_) contributions.

Assuming the presence of a coherent single-L2_1_-precipitate, the dependence of the strength increase on the precipitate size has been evaluated using Eqs [1, 2, 3], as shown in Fig. 7, based on APB energy (*γ*_*APB*_ = 0.058 J/m^2^) of the L2_1_-Ni_2_TiAl for the (110) plane^42^, Δ*G* = 22.0 GPa, which is determined by the elastic constants of the FEM modeling in Table 2, and *δ* = 0.0077 for HPSFA at 973 K, determined from the current ND experiments (Table 1). As can be seen, the strength increase from the shearing contributions increases with increasing the size of the precipitate. The strength increase of the shearing contributions from the current calculation is expected to be under-estimated, since the precipitate of HPSFA is a two-phase coupled-structure of the B2 and L2_1_. Furthermore, the APB energy of the B2 for the (110) plane is 0.5J/m^2^, which is higher than that of the L2_1_ phase^42^. Therefore, the actual strength increase curve of the shearing mechanism is believed to be higher than the calculated curve, which is much higher than the experimental value of the yield strength of HPSFA and SPSFA. These trends indicate that the precipitate shearing is unlikely to happen in the current alloys.

Another feasible mechanism is the Orowan dislocation bypass mechanism^43^,^44^, which is defined as


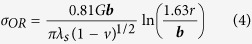



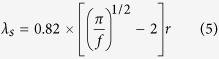


where *λ*_*s*_ is the square lattice spacing and *v* = 0.3 is the Poisson’s ratio. Based on the volume fractions of the precipitate for the studied alloys (*f* = 16% for HPSFA and 22% for SPSFA^18^), two curves of Orowan strength contribution with respective to the precipitate size were calculated using Eqs [4, 5] and also included in Fig. 7. Good agreement is observed between the experimental and theoretical values. Generally, in Orowan-bowing mechanism, dislocations pass through the inter-particle region, and dislocation loops are created around precipitates. It can be inferred that the observed increase of the precipitate strain in Fig. 5 is caused by the dislocation array around the precipitates, which accommodate the lattice misfit between the matrix and precipitate. Therefore, it is concluded that the Orowan dislocation bypass is the dominant mechanism for HPSFA and SPSFA at 973 K.

### Effect of Interface Structure on Mechanical Properties

The evolution of the elastic strain of constitutive phases in HPSFA and SPSFA shows the qualitative agreement with the macroscopic stress-strain behavior, and can provide an in-depth understanding of the deformation mechanisms of the current alloys at high-temperatures. The beginning of the load transfer from the matrix to precipitate is indicative of the onset of the macroscopic yielding behavior. The strain hardening behavior is also accompanied by the increase in elastic strain of (or the load carried by) the precipitate, which reflects the significance of the load-transfer effectiveness of the precipitate on the mechanical properties of the current alloys at high temperatures. Furthermore, HPSFA with the coherent precipitate shows the greater load-transfer capability, such as the maximum value of the elastic strain of the precipitates, and strain-hardening capability of the matrix, than SPSFA with the semi-coherent precipitate. Therefore, the interface structures of the precipitates are believed to play a critical role in improving the load-transfer capability, and, thus, the mechanical properties at high temperatures.

**Figure 8:**
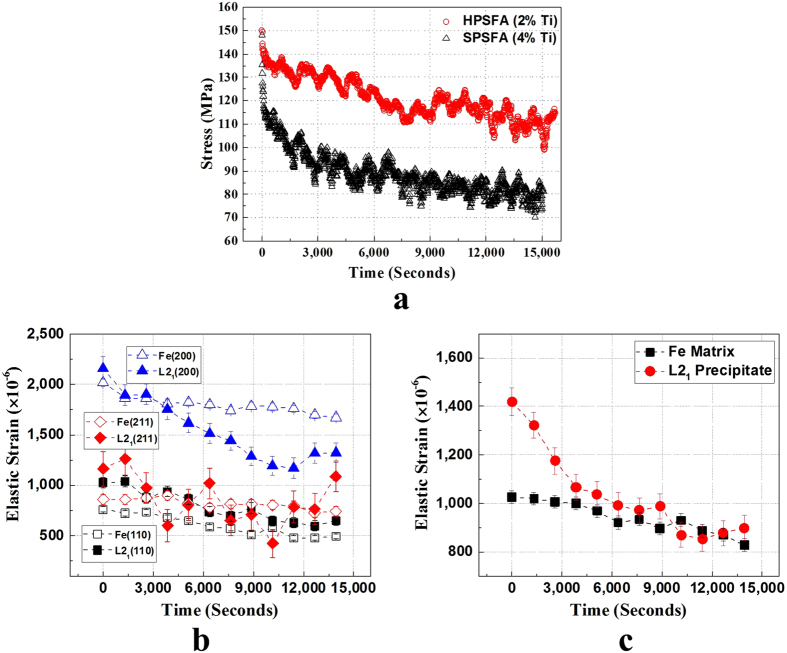
Temporal evolution of stress relaxation. (**a**) Temporal evolution of the macroscopic stress for SPSFA and HPSFA, and evolution of the elastic strain of the Fe and L2_1_ phases, derived from (**b**) single-peak and (**c**) whole-peak fitting approaches, during the stress relaxation of SPSFA at 973 K.

During the ND data measurement at 973 K, a certain amount of stress relaxation occurs for both alloys (Fig. 4). The stress relaxation at high temperatures has been studied to understand deformation mechanisms at high-temperatures, such as creep mechanisms^32^,^33^. The kinetics of this stress relaxation is known to be largely governed by dislocation processes and possibly grain boundary sliding in materials^32^. In the present alloys, the grain size of HPSFA and SPSFA are estimated to be larger than 200 μm, and, thus, the stress relaxation caused by the grain-boundary sliding is not expected to be significant. Instead, the phase-interface between the matrix and precipitate could play an important role in the stress relaxation, and, hence, the load-transfer between the constitutive phases at high temperatures. However, the holding modes applied during the relaxation are slightly different [actuator displacement control in Fig. 3(a), and strain gauge control in Fig. 3(b)], which could result in the different relaxation behavior. Therefore, to verify this hypothesis, we conducted stress relaxation tests on the HPSFA and SPSFA at 973 K. The samples were loaded to 150 MPa at 973 K and held in displacement holding mode for about 4 hours. The ND data was collected for the SPSFA in every 20 mins, and the ND data was analyzed using the Rietveld whole-peak fitting and single fitting approaches using the GSAS program developed at the Los Alamos National Laboratory^30^ to obtain the averaged lattice parameters and (hkl) plane spacing of the Fe and L2_1_ phases, respectively. The temporal evolution of the stress relaxation and the elastic strain at 973 K for SPSFA are displayed in Fig. 8.

The macroscopic stress-relaxation curves in Fig. 8a exhibits the logarithmic decay of the stress and the level of the stress reduction of HPSFA is lower than that of the SPSFA, which is consistent with the relaxation behavior in Fig. 4. The residual stress ratio is introduced to characterize the stress relaxation resistance and relaxation efficiency. The residual stress ratio (ψ) is defined as follows^45^,


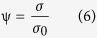


where *σ*_0_ and *σ* are the initial stress and residual stress at a certain time, respectively. The stress residual ratios of HPSFA and SPSFA are calculated using Eq. [6] and are 67.5% and 47.5% at 973 K, respectively. These results clearly demonstrate that the level of the stress relaxation of SPSFA is more significant than that of HPSFA, as observed in Fig. 4.

The ND data measured during the relaxation of SPSFA were analyzed using the single-peak and whole-peak fitting approaches to determine the averaged lattice parameters and (hkl) plane spacings of the constituent phases, respectively. The elastic strains during the relaxation was obtained using Eq. (7), and elastic strain evolutions derived from the single-peak and whole-peak fitting are presented in Fig. 8(b) and (c), respectively. The evolution of the elastic strain obtained from the single-peak fitting is well consistent with that obtained from the whole-peak fitting, revealing more considerable reduction of the elastic strain of the precipitate, as compared to that of the matrix. Due to the huge grain size, the slight decreases of the Fe matrix lattice strain with increasing the relaxation time is not expected to result from the grain-boundary sliding or diffusion, at given the relatively-low homologous temperature and high stresses. The decrease of the Fe matrix strain may be due to a combination of dislocation and diffusional creep^18^. More importantly, as the relaxation time increases, the elastic strain of the precipitate is rapidly reduced, and becomes comparable to that of the Fe matrix until there is no load transferred to the precipitate from the plastically-deforming matrix. These results strongly support that the elastic strain of the precipitate, or equivalently the load transferred to the precipitate, is released by the diffusional process along the interface between the matrix and precipitate. It can be inferred that dislocations are pinned around the precipitates (such as Orowan dislocation loops), which accommodates the lattice misfit created by plastic-elastic strain anisotropy between the matrix and precipitate. During the holding, the pinned dislocations can be annihilated via diffusional flow or bypass the precipitate via dislocation climb mechanism. It has been reported that the deformation of the initial and transition regimes of the stress relaxation is dominated by the dislocation climb bypassing mechanism^33^. Therefore, in the current study, it is believed that the stress relaxation is mainly affected by the diffusional process (annihilation or bypass of dislocations) along the interface between the matrix and precipitate.

The coherent hierarchical precipitate of HPSFA exhibits the more pronounced load-transfer and strain hardening capability than the semi-coherent single precipitate of SPSFA, which reflects the effectiveness of the coherent-precipitate structure. The coherency strain field in the Fe matrix, reinforced by the hierarchical-precipitate structure, could be effective in enhancing the matrix hardening capability via the reduced diffusional flow along the interface between precipitate and matrix, thus, the strain-hardening and strengthening behavior of HPSFA at high temperatures. The efficiency of load-transfer from the matrix and precipitate as a function of temperature has been investigated using *in*-*situ* ND experiments for the NiAl-strengthened ferritic alloy^31^. It was reported that no load transfer was observed between the Fe matrix and NiAl precipitate at 973 K. It was explained by the fact that diffusional flows along the matrix–precipitate interface can relax the interphase strain, leading to ineffective load transfer from the matrix to the precipitate phases and accelerated creep deformation. Moreover, the precipitate in SPSFA contains a high density of misfit dislocations that can reduce the elastic strain in the Fe matrix^18^. The misfit dislocations could allow for the pipe diffusion, which can increase the creep deformation rate. Thus, the interface elastic-strain field is believed to have significant effect on the high-temperature mechanical properties of coherent-precipitate-strengthened materials^37^,^46^,^47^. In this regard, our results suggest that the hierarchical-precipitate alloy-design strategy provide a novel way to enhance the elastic strain field in the matrix and improve the mechanical properties at high temperatures.

Overall, the deformation behavior of Fe-Cr-Ni-Al-Ti alloys strengthened by hierarchical Ni_2_TiAl/NiAl and single Ni_2_TiAl precipitates has been studied at 973 K. The hierarchical precipitate-strengthened alloy with 2-wt.% Ti (HFSFA) contains a coherent hierarchical precipitate with a two-phase coupled structure of the B2-NiAl and L2_1_-Ni_2_TiAl phases, while the single precipitate-strengthened alloy with 4-wt.% Ti (SPSFA) is reinforced by a semi-coherent single-L2_1_-Ni_2_TiAl precipitate with a high density of misfit dislocations. The *in*-*situ* tension ND experiments on both alloys at 973 K were conducted to study the evolution of lattice strains at phase levels in combination with crystal-plasticity finite-element model. The macroscopic stress-strain behavior is qualitatively consistent with the evolution of the phase strains, showing the interphase load transfer from the matrix to precipitate in plastic regime. The macroscopic yield-strength at 973 K is in quantitative agreement with stress values evaluated by classical dispersion-strengthening theory, suggesting Orowan dislocation bypass to be the governing deformation mechanism for both alloys at 973 K. The crystal-plasticity finite-element model shows qualitative agreement with the experimental results until softening occurs where the flow stress gradually decreases with increasing plastic strain, reflecting the significance of the diffusional process on the deformation behavior at 973 K. Relaxation tests on HPSFA and SPSFA was carried out with *in*-*situ* neutron diffraction. These results revealed the gradual decrease in the precipitate strain transferred from the plastically-deforming matrix, as the relaxation time increases, which supports that the load transfer capability at high temperatures is strongly affected by the diffusional flow along the interface between the matrix and precipitate. The *in*-*situ* tension tests at 973 K shows the higher level of the stress relaxation for SPSFA during the ND measurements than that for HPSFA, indicating the insufficient diffusional flow in HPSFA with the coherent precipitate structure, relative to SPSFA with the semi-coherent precipitate. The load transfer capability of HPSFA and SPSFA is found to be related to the precipitate interface structures, such as coherent strain field in the matrix, created by the lattice misfit between the matrix and precipitate. The coherent strain field in the matrix of HPSFA plays an important role in enhancing the load-transfer capability and strengthening behavior of the material at high temperatures. These results could provide a new alloy-design strategy, accelerate the advance in the development of elevated-temperature engineering materials, and broaden the applications of ferritic alloys to higher temperatures.

## Methods

### Materials and Microstructural Characterization

The nominal composition of the alloys is Fe-6.5Al-10Cr-10Ni-xTi-3.4Mo-0.25Zr-0.005B with x = 2 and 4 in weight percent (wt. %). A plate ingot of the 2-wt.%-Ti alloy with a dimension of 12.7 × 25.4 × 1.9 cm^3^ and a rod ingot of the 4 wt.%-Ti alloy with 2 Kg and a diameter of 5.08 cm were prepared by the Sophisticated Alloys, Inc., using the vacuum-induction-melting facility. Hot isostatic pressing (HIP) was applied to the ingots at 1,473 K and 100 MPa for 4 hours in order to reduce defects formed during the casting and cooling processes. Chemical analyses were conducted on the ingot to obtain the bulk composition. These alloys were homogenized at 1,473 K for 30 minutes, followed by air cooling and, then, aged at 973 K for 100 hours. The thin foils for conventional transmission-electron-microscopy (CTEM) observations were prepared by electropolishing, followed by ion milling at the ion energy of ~2 kV and an incident angle of ±6 degree. The TEM specimens were cooled by liquid N_2_ during ion milling. The TEM observations were conducted with a Zeiss Libra 200 MC TEM/STEM. TEM images were acquired at an acceleration voltage of 200 kV.

### *In*-*situ* Neutron Diffraction Experiments

The *in*-*situ* neutron diffraction (ND) experiments on 2-wt.%-Ti and 4-wt.%-Ti alloys were carried out on the Spectrometer for MAterials Research at Temperature and Stress (SMARTS) diffractometer of the Los Alamos Neutron Science Center (LANSCE) facility located at the Los Alamos National Laboratory, United State^48^ and ENGIN-X facility located at the ISIS, United Kingdom^49^, respectively. The ND diffractometers utilize time-of-flight (TOF) measurements, in which the incident beam is polychromatic with a range of wave lengths, which allows for the ND measurements with a diffraction pattern covering a wide range of d spacings without the rotation of samples or detectors. Two detectors, which are fixed at an angle of 45° to the loading direction, were employed to collect the diffracted beams from polycrystalline grains with lattice planes parallel to the transverse and axial directions, respectively. Therefore, the lattice parameters of the constitutive phases can be measured simultaneously both parallel and perpendicular to the loading directions. Screw-threaded cylindrical samples with a gage diameter of 6.35 mm and a gage length of 40 mm were machined for the *in*-*situ* loading experiments at LANSCE, while ones with a gage diameter of 8 mm and a gage length of 42 mm at ENGIN-X. The ND measurements were conducted at room and elevated temperatures up to 973 K. The ND data for 2 and 4-wt.%-Ti alloys were collected for about 12 and 20 minutes, respectively. The *in*-*situ* tension experiment at LANSCE was carried out in vacuum at 973 K, while that at ENGIN-X was conducted in air. In addition to the *in*-*situ* tension experiments, stress relaxation tests on HPSFA and SPSFA at 973 K were carried out at ENGIN-X. The screw-threaded cylindrical samples were loaded to 150 MPa at 973 K and held in displacement holding mode for about 4 hours. The ND measurements were able to be made only on SPSFA in every 20 mins due to the neutron interruption during the ND measurements on HPSFA. The macroscopic strain was measured using a high-temperature extensometer over a gauge length of 10 mm within a measureable strain range of 10%.

### Neutron Data Analysis

Our ND analysis was conducted under an assumption that the diffraction intensity from the precipitate mainly originates from the L2_1_-structure phase, and one from the low volume fraction of B2-structure phase does not appreciably contribute to the diffraction intensity. The lattice parameters of the Fe and L2_1_ phases were determined from a whole-pattern Rietveld refinement employing the GSAS program developed at the Los Alamos National Laboratory^30^. The ND data during the heating and entire *in*-*situ* loading experiments can be successfully refined with the value of χ^2^ ranging from 1 to 2, which gives a confidence on our analysis.

The averaged phase strain represents the volume-averaged lattice strain of the individual phases (α-Fe and Ni_2_TiAl), which depends on the elastic and plastic anisotropy of the individual phases. The average phase strain is calculated, using the following formula


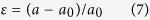


where *a* is the lattice parameter or plane spacing of a given phase measured during heating and/or loading, and *a*_0_ is the corresponding lattice parameter or plane spacing before loading (5 MPa at 973 K). The lattice parameters determined from the Rietveld refinement approach were utilized for the misfit calculations, which is defined as


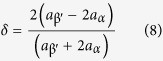


where *δ* is the lattice misfit, and *a*_*α*_ and *a*_β′_ are the lattice parameters of α-Fe and Ni_2_TiAl phases, respectively. Since the L2_1_ structure consists of eight sub-lattices of a B2 structure^50^, the lattice parameter of the Fe was multiplied by a factor of two for the corresponding structural comparison.

### Finite-Element Crystal-Plasticity Model

The microstructure-based finite-element simulations use the crystal-plasticity model, which is implemented as a user material subroutine in the commercial software ABAQUS^51^. The constitutive relationship used in the simulations was defined through the Peirce-Asaro-Needleman power law, which relates the slip rate and the resolved shear stress on a given slip system by,


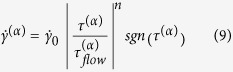


The flow stress increases as a function of the cumulative slip strains,


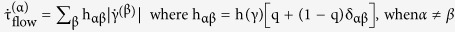


However, for the self-hardening model, we use,


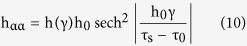


where h_0_ is the initial hardening modulus, τ_0_ is the initial slip strength, and τ_s_ is the saturated slip strength. 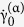 is the characteristic strain rate, n is the stress component, and 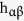 is the latent hardening moduli. The terms, τ^(α)^ and 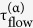, are the resolved shear stress and flow strength of the α-th slip system, respectively, and q is the latent-hardening coefficient in the same set of slip systems.

**Figure 9:**
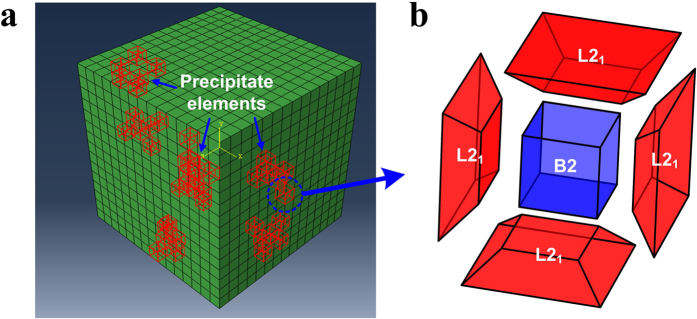
Elemental cubic model for CP-FEM. (**a**) Schematic illustration of a 15 × 15 × 15 elements cubic model, employed in the simulation of HPSFA, and (**b**) the detailed structure of a modeled precipitate element consisting of 6 trapezoidal elements of the L2_1_ phase (only 4 elements are shown for better visualization) and a centered cubic element of the B2 phase.

For the 2-wt.%-Ti alloy with the hierarchical precipitate consisting of the B2 and L2_1_ phases, a cubic model with 15 × 15 × 15 elements was set up (Fig. 9a), and divided into 125 grains with each of 27 elements making up a 3 × 3 × 3 cubic grain. The 5 elements were randomly selected and assumed to be the L2_1_ precipitates in each grain. The volume fraction of the constitutive phases has been previously determined using SEM, TEM, and APT techniques^18^,^19^. Firstly, the SEM images were analyzed using the ImageJ software to estimate the volume fraction of the SPSFA and HPSFA in the different heat-treatment conditions^18^. Secondly, the chemistry of the constituent phases obtained from the TEM and APT was used to estimate the volume fraction based on the lever rule^19^. Lastly, based on the chemistry of the phases and the corresponding structure factors, the neutron diffraction results were quantitatively analyzed to estimate the volume fraction^19^. These results show consistent volume fraction ranging from 16~22 vol. %. Based on these results, the volume fraction of the L2_1_ precipitates, compared to the Fe matrix, was initially set to be 18.5%. A second type of B2 precipitates was introduced with an element smaller in size embedded in the pre-existing L2_1_ elements in each grain, as shown in Fig. 9b. The B2 elements were set to have a volume fraction of 50%, compared to L2_1_ elements, and 9.25%, relative to the Fe matrix. In essence, each 27-element grain contains a total of 22 elements assigned to the Fe matrix crystal-plasticity parameters. Each of the remaining 5 elements contains 6 trapezoidal elements at the 6 faces of the cubic element with a smaller cubic elements attached at the center of these 5 elements. The trapezoidal elements were assumed to have the L2_1_-precipitate properties, and the smaller cubic elements were assumed to have B2-precipitate properties. For the 4-wt.%-Ti alloy with the single L2_1_ precipitate, A total of 125 (3 × 3 × 3) randomly-oriented cubic grains were used in the model. Each grain consists of 27 elements, and 6 of them were assigned to be L2_1_-type precipitate.

The values of elastic constants (C_11_, C_12_, and C_44_) of the Fe matrix, L2_1_, and B2 phases at 973 K from References^18^,^31^ were initially employed, and further adjusted by comparing with experimental diffraction elastic constants. Precipitate elements were set to deform elastically, and, hence, the elastic constants of the precipitate are considered as only variables in the modeling parameters. Other parameters, such as *h*_0_, *τ*_*s*_, and *τ*_0_, for the Fe matrix, which determine the plastic deformation behavior, are only tuned so that the modelled stress vs. elastic phase-strain curves follow the experimental results. It is known that the slip strength (*τ*_0_), is related to the macroscopic yield strength of a polycrystal by the Taylor factor, which is about 3 for a bcc material. All the parameters employed in the current model are listed in Table 2.

## Additional Information

**How to cite this article:** Song, G. *et al*. High Temperature Deformation Mechanism in Hierarchical and Single Precipitate Strengthened Ferritic Alloys by In Situ Neutron Diffraction Studies. *Sci. Rep.* **7**, 45965; doi: 10.1038/srep45965 (2017).

**Publisher's note:** Springer Nature remains neutral with regard to jurisdictional claims in published maps and institutional affiliations.

## Figures and Tables

**Table 1:** Lattice parameters and misfits.

Temperature (K)	4-wt.%-Ti alloy			2-wt.%-Ti alloy		
	α-Fe (Å)	L2_1_ (Å)	Misfit (%)	α-Fe (Å)	L2_1_ (Å)	Misfit (%)
300	2.88950 ± 0.000044	5.85269 ± 0.000182	1.26 ± 0.30	2.88851 ± 0.000027	5.80566 ± 0.000591	0.49 ± 0.19
973	2.91921 ± 0.000044	5.91624 ± 0.000182	1.32 ± 0.31	2.91507 ± 0.000037	5.87570 ± 0.000682	0.77 ± 0.28

Summary of lattice parameters and misfit between the Fe matrix and L2_1_ phases for the 2 and 4-wt.%-Ti alloys at room temperature and 973 K.

**Table 2:** Summary of parameters employed in the elastic-plastic constitutive law.

Material	Phase	C_11_ (MPa)	C_12_ (MPa)	C_44_ (MPa)	τ_0_ (MPa)	τ_s_ (MPa)	n	h_0_	q
4-wt.%	Fe	160,000	128,000	92,000	35	157	10	100	1
	L2_1_	120,000	97,000	56,000	>200	—	—	—	—
2-wt.%	Fe	160,000	128,000	92,000	50	157	10	500	1
	L2_1_	120,000	97,000	56,000	>200	—	—	—	—
	B2	130,000	106,000	70,000	>200	—	—	—	—

C_11_, C_12_, and C_44_: Elastic constants, n: stress component, q: latent-hardening coefficient in the same set of slip systems, h_0_: initial hardening modulus, τ_s_: saturated slip strength, and τ_0_: initial slip strength.
